# Endoscopic incision of malignant stenosis for the retrieval of a retained capsule endoscope

**DOI:** 10.1055/a-2240-9070

**Published:** 2024-02-07

**Authors:** Takashi Taida, Ryosuke Horio, Kenichiro Okimoto, Yuki Ohta, Tomoaki Matsumura, Jun Kato, Naoya Kato

**Affiliations:** 1 Gastroenterology, Chiba University Graduate School of Medicine, Chiba, Japan; 2 92154 Endoscopic Center, Chiba University Hospital, Chiba, Japan

Capsule endoscopy is one of the least invasive options for enteroscopy, allowing detailed observation of the entire small intestine. However, capsule retention can occur, particularly in patients with small-intestinal stenosis, which may require surgical retrieval in some cases 1 . In cases of capsule retention due to small-intestinal stenosis in Crohn’s disease or other benign lesions, retrieval using balloon enteroscopy has recently been performed. Endoscopic retrieval involving stenosis dilation has also been reported 2 3 4 . Here, we report a case of successful endoscopic retrieval of a capsule endoscope retained in a malignant tumor through an incision made using a needle-knife ( Video 1 ).

Endoscopic retrieval of a capsule endoscope retained in a malignant tumor through an incision made using a needle-knife.Video 1

A 73-year-old man underwent capsule endoscopy for suspected small-intestinal bleeding. Capsule endoscopy revealed multiple ulcers at the distal end of the ileum, indicating a neoplastic lesion. Abdominal X-ray and computed tomography (CT) scan showed a tumor at the location and the capsule retained in the tumor area due to abnormal thickening of the intestinal wall ( Fig. 1 ). Transanal double-balloon small-bowel enteroscopy (EN-580T; Fujifilm Medical, Tokyo, Japan) was performed to remove the capsule, but the capsule was retained at the oral side of the stenosis caused by the malignant tumor. The tumor was diagnosed as malignant lymphoma based on the biopsy specimen. Because the capsule could not pass through the stenosis due to the tumor protruding into the lumen, the tumor was incised using a needle-knife ( Fig. 2 ), allowing the capsule to be successfully pulled out through the incised tumor ( Fig. 3 ). The incision was accompanied with slight hemorrhage, but no other complications occurred. Endoscopic incision using balloon enteroscopy can be used to remove a retained capsule endoscope due to a small bowel malignant tumor.

**Fig. 1:**
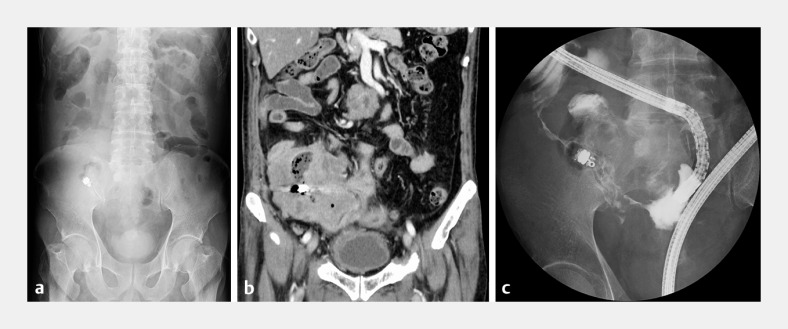
Retained capsule endoscope. **a** Abdominal X-ray showing a retained capsule endoscope in the right lower part of the abdomen. **b** Abdominal computed tomography (CT) showing a large malignant tumor located adjacent to the retained capsule. **c** Enteroscopy showing luminal stenosis and the retained capsule located beyond the stenosis.

**Fig. 2:**
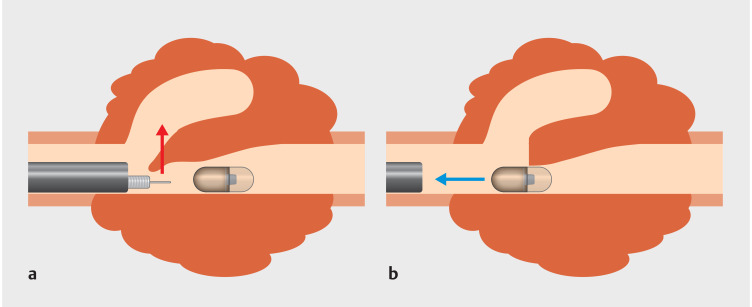
Illustration of capsule retrieval through the endoscopic incision. **a** The protruded tumor was incised using a needle-knife. **b** The retained capsule was successfully retrieved.

**Fig. 3:**
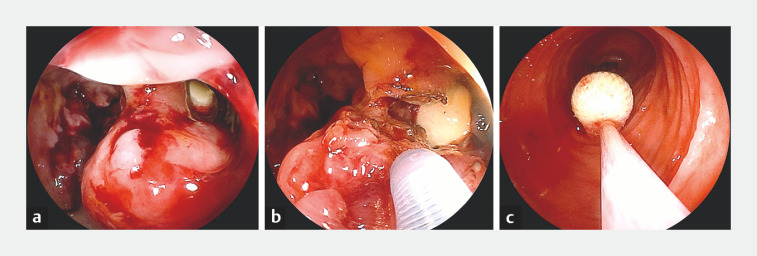
Endoscopic incision of malignant stenosis. **a** Retained capsule is visible at the oral side of the malignant stenosis. **b** The malignant stenosis was incised with a needle-knife. **c** The capsule was successfully removed endoscopically.

Endoscopy_UCTN_Code_TTT_1AQ_2AF

## Notes

Endoscopy E-Videos https://eref.thieme.de/e-videos
*E-Videos* is an open access online section of the journal *Endoscopy* , reporting on interesting cases and new techniques in gastroenterological endoscopy. All papers include a high-quality video and are published with a Creative Commons CC-BY license. Endoscopy E-Videos qualify for HINARI discounts and waivers and eligibility is automatically checked during the submission process. We grant 100% waivers to articles whose corresponding authors are based in Group A countries and 50% waivers to those who are based in Group B countries as classified by Research4Life (see: https://www.research4life.org/access/eligibility/ ). This section has its own submission website at https://mc.manuscriptcentral.com/e-videos .
